# DNA methylation and de-methylation using hybrid site-targeting proteins

**DOI:** 10.1186/s13059-018-1566-2

**Published:** 2018-11-06

**Authors:** Yong Lei, Yung-Hsin Huang, Margaret A. Goodell

**Affiliations:** 1 Stem Cells and Regenerative Medicine Center, Baylor College of Medicine, Houston, TX 77030 USA; 2 Center for Cell and Gene Therapy, Baylor College of Medicine, Houston, TX 77030 USA; 3 Program in Developmental Biology, Baylor College of Medicine, Houston, TX 77030 USA; 4 Department of Molecular and Human Genetics, Baylor College of Medicine, Houston, TX 77030 USA

## Abstract

DNA methylation plays important roles in determining cellular identity, disease, and environmental responses, but little is known about the mechanisms that drive methylation changes during cellular differentiation and tumorigenesis. Meanwhile, the causal relationship between DNA methylation and transcription remains incompletely understood. Recently developed targeted DNA methylation manipulation tools can address these gaps in knowledge, leading to new insights into how methylation governs gene expression. Here, we summarize technological developments in the DNA methylation editing field and discuss the remaining challenges facing current tools, as well as potential future directions.

## Introduction

DNA methylation is a covalent modification of DNA that is involved in many biological processes, including transcription regulation, genomic imprinting, X-chromosome inactivation, and loss of pluripotency [1–6]. Aberrant DNA methylation has also been observed in multiple cancers, aging, and neurodegenerative diseases [7, 8]. Despite more than 20 years of research on the dynamics of DNA methylation and its effect on gene expression during development and disease initiation, many important questions remain. Our current understanding of the roles of DNA methylation is informed predominantly by techniques that are based on the removal of DNA epigenetic modifier enzymes and the study of patients with epigenetic modifier mutations. To date, few studies have utilized site-specific manipulation of DNA methylation to add and remove specific epigenetic marks in order to shed light on the regulatory roles of methylation in disease and development.

Large-scale projects such as the Encyclopedia of DNA elements (ENCODE) and Roadmap Epigenomics have provided sizable data sets for analysis and interpretation. The lack of highly specific and efficient tools for DNA methylation and demethylation has been the bottleneck for dissecting the role of DNA methylation further. Recent advances in targeted DNA cleavage technologies, including protein-based zinc-finger (ZF) nucleases (ZFNs), transcription activator-like effector nucleases (TALENs), and the RNA-guided CRISPR-Cas9 system, have enabled the development of customizable targeted DNA recognition platforms [9]. In order to edit targeted cytosine methylation at specific sites, most studies to date have coupled anchoring platforms listed above with a methylation writer/eraser protein. These tools are being used to address how DNA methylation affects local and distal regulatory elements in gene transcription, as well as the causal relationship between DNA methylation and transcription.

Here, we focus on recent advances in targeted DNA methylation and demethylation tools, and discuss the insight they have provided into how forcible methylation and demethylation govern gene expression. Finally, we discuss current challenges and potential improvements to these tools in relation to their wider application in the field.

## Milestones in the development of targeted DNA methylation tools

**Fig. 1:**
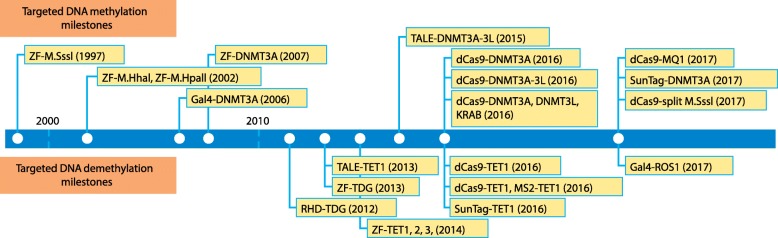
The development of targeted DNA methylation and demethylation milestone tools. Not drawn to scale

More than 20 years ago, the silencing of gene expression using targeted methylation with a fusion protein was reported for the first time. In this pioneer study, the authors used bacterial cytosine-5 methyltransferase M.SssI and ZF triplicate protein (Zip53-M.SssI, Fig. 1) to methylate the p53-binding site at the *p21*^*WAF1/CIP1*^ gene [10]. Additional prokaryotic DNA methyltransferases, including M.Hhal and M.HpaII [11, 12], were subsequently used for targeted DNA methylation. However, these tools caused strong off-target effects [11–13], potentially because of the non-specific binding of ZFs [14, 15]. Further study demonstrated that methylated DNA itself attenuates the binding affinity of ZFs, limiting the epigenetic application of this strategy [16]. Nevertheless, customized ZF-based chimeric DNA methyltransferases have been applied widely [17–29]. Aside from targeted CpG methylation, the catalytic domain of M.CviPI, a DNA methyltransferase recognizing GC sites, was fused to the yeast trans-activator Pho4 in order to induce methylation at the *PHO5* promoter [30].

In 2009, transcription activator-like effectors (TALEs) were reported as second-generation DNA-binding platforms that made the design and construction process of targeted DNA methylation tools more straightforward and less labor-intensive [31, 32]. Unlike ZFs, which recognize DNA nucleotide triplets, each TALE domain recognizes and binds a single nucleotide. Moreover, there is minimal cross-talk between adjacent TALE modules, making the interaction between TALE domain and targeted sequences less complex than similar interactions involving ZFs. Bernstein et al. [33] first applied chimeric TALE modules involving the catalytic domain of the DNA methyltransferases DNMT3A and DNMT3L (TALE–DNMT3ACD–3 L) to introduce methylation at the *CDKN2A* locus and to decrease gene expression in fibroblasts (Fig. 1). Recently, an optogenetically controlled fusion protein of TALE and the catalytic domain of DNMT3A (TALE–DNMT3ACD) was used to edit the *Ascl1* gene in neural stem cells [34]. However, DNA methylation was found to weaken the anchoring of TALE at 5-mC sites, similar to results reported for ZFs. Published studies demonstrated that TALE-based tools still require special designs to achieve maximum binding activity [35, 36].

The advent of CRISPR-based tools offered a new, more versatile approach. The ability of CRISPR-Cas9 to target many different sites using multiple guide RNAs (sgRNAs) has led to its widespread use for targeted activation, repression, and epigenetic editing [37]. In epigenetic editing, dCas9 (catalytic dysfunction Cas9) is often used as a binding platform. Vojta et al. [38] first fused dCas9 with the catalytic domain of DNMT3A (dCas9–DNMT3ACD) to target the *BACH2* and *IL6ST* loci in human embryonic kidney cells (HEK293T). Using this tool, the authors achieved an increase of up to 60% CpG methylation at the *BACH2* locus. A similar study by McDonald et al. [39] demonstrated an increase of up to 50% in DNA methylation at the *CDKN2A* and *ARF* loci using multiple sgRNAs. Significant off-target methylation using non-specific sgRNAs was also observed. Instead of utilizing the DNMT3A catalytic domain, Liu et al. [40] fused full-?>length DNMT3A protein with dCas9 (dCas9–DNMT3A) and successfully induced targeted CpG methylation both in vitro and in vivo. Using quantitative chromosome conformation capture (3C), these authors further demonstrated that the targeted methylation of CTCF-binding sites (CBS) alters local looping and transcription at both *mi290* and *Pou5f1* loci [40]. These studies showed that hybrid DNA methylation tools enable efficient editing in a local manner.

Previous reports have shown that DNMT3L enhances de novo methylation activity by forming hetero-tetramers with the catalytic domain of DNMT3A [41, 42], and thus could potentially be used to improve current DNA methylation editing strategies. Amabile et al. [43] showed heritable and stimulation-resistant silencing of endogenous genes by co-delivery of combinations of dCas9-based engineered transcription repressors (ETRs), including DNMT3ACD, DNMT3L, and Krupple-associated box (KRAB). Another study, from Stepper et al. [44], showed that a single sgRNA guided dCas9–DNMT3ACD–3 L provided higher levels of DNA methylation than dCas9–DNMT3ACD at three individual promoters. Further analysis of the distribution of DNA methylation indicated that entire CpG islands (CGI) (around ±400 bp around the protospacer adjacent motif (PAM)) could be effectively methylated using a single sgRNA.

Multimerization of transcription factors plays an important role in many biological processes. Building on this principle in order to convey transcriptional activation, Tanenbaum et al. [45] invented a SunTag strategy, which applied a modified dCas9-binding platform to recruit up to 24 copies of the intended protein via a repeating peptide array at targeted loci. A further study proved that this approach could be adapted for targeted DNA demethylation [46] (as discussed below). By adopting this strategy, Huang et al. [47] showed that dCas9–SunTag–DNMT3A recruited multiple DNMT3As to the desired sites and hypermethylated CpGs in a region of up to 4.5 kb at *HOXA* loci. Thus, applying SunTag to methylation and demethylation tools is one promising strategy for long-range methylation editing.

**Fig. 2:**
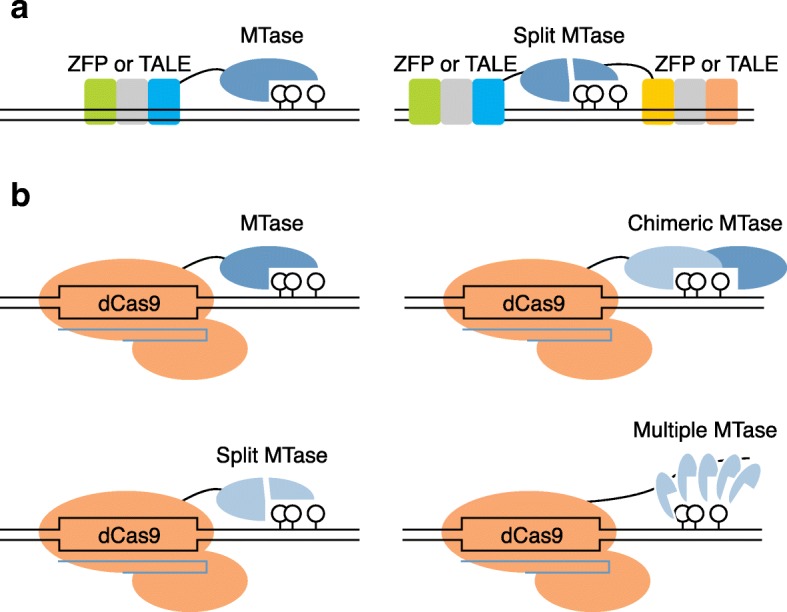
Current strategies of targeted CpG methylation. **a** Main strategies of zinc-finger proteins (*ZFP*) or transcription activator-like effector (*TALE*)-associated hybrid proteins. Methyltransferase (*MTase*) can be fused to a ZFP or TALE anchoring platform with or without a linker. Split MTase can be fused to a ZFP or TALE pair to improve binding specificity. **b** Main strategies for the use of CRISPR-based hybrid proteins. MTase can be fused to dCas9 with or without a linker. Chimeric MTase such as DNMT3A–DNMT3L has been applied to increase methylation. Split MTase (M.SssI) can decrease off-target effects. MTase multimerization approaches (such as dCas9–SunTag) enhance activity for long-range methylation editing

One drawback of the tools described above is the amount of time necessary for their application. To achieve more rapid methylation, the dCas9–MQ1^Q147L^ fusion protein (derived from *Mollicutes spiroplasma* M.SssI) was introduced to generate targeted methylation effectively within 24 h [48]. In this study, targeted de novo methylation at the mouse *Igf2/H19* imprinting loci was achieved by a direct zygote injection strategy. The rapid editing activity of dCas9–MQ1^Q147L^ makes this tool potentially applicable to the study of early embryogenesis. In order to improve specificity, a recent study split the M.SssI methyltransferase into two parts (N-terminal and C-terminal). The authors then fused the C-terminus to dCas9 to guide the functional assembly of the methyltransferase to targeted CpGs, providing a new strategy to enable precise and multiplex control over CpG methylation [49]. Thus, numerous tools for targeted manipulation of CpG methylation using different DNA-binding platforms and methyltransferases, each with specific strengths and weaknesses discussed in more detail below, have been applied successfully in vitro and in vivo (Fig. 2).

## Milestones in the development of targeted DNA demethylation tools

In comparison with targeted DNA methylation, tools to induce targeted DNA demethylation have a shorter history, probably in part because there is no single mechanism to remove methylation directly in mammals. Active removal of 5mC demethylation involves iterative oxidation and thus requires multiple steps. In one pioneering study, Gregory et al. [50] selected the Rel-homology domain (RHD), a well-characterized NFkB-binding domain, to anchor thymine DNA glycosylase (TDG) via a short glycine-rich linker. These authors observed a loss of DNA methylation at the targeted locus as well as increased transcription of *Nos2* in the NIH3T3 cell line. In order to increase the flexibility of targeting, the group replaced RHD with ZFs. Using whole-genome expression microarrays and pathway analysis, the authors found that the targeted demethylation of *Nos2* affected only 42 genes, and that the majority of these genes were downstream of *Nos2* [51]. These studies demonstrate that targeted DNA demethylation using TDG can upregulate gene expression.

Ten-eleven translocation (TET) enzymes participate in the initial step of DNA demethylation by oxidizing 5mC to 5hmC, which can lead to demethylation. The TET hydroxylase catalytic domain can also be utilized for targeted DNA demethylation. Both ZF and TALE have been used as anchoring platforms for TET enzymes, and both systems were able to increase transcription at targeted loci [52, 53]. Although ZF–TET led to no obvious off-target effects at LINE-1 elements when assessed using pyrosequencing [52], the TALE–TET1 study reported marginal off-target demethylation, which was possibly induced by the hydroxylase catalytic domain. Non-specific TALE binding is unlikely to be responsible for the off-target effects that have been observed [53]. Moreover, based on the fact that some CpGs are more efficiently demethylated than others, Maeder et al. [53] hypothesized that the extent of the demethylation observed may represent a steady state between demethylation and re-methylation. To support this hypothesis, the authors showed significant re-methylation as TALE–TET1-encoding plasmid was lost from cells. Similar dynamics were also reported in attempts to achieve targeted CpG methylation in which levels of methylation at desired sites decreased after reaching peak efficiency in a time-dependent manner [38, 39, 48, 54, 55]. Thus, additional cellular factors, including histone modification and chromosome accessibility, probably participate in enforcing the DNA status after methylation is introduced.

Of note, in the study using ZF–TET, Chen et al. [52] compared the demethylation activity of the catalytic domains of TET1, TET2, and TET3. These authors showed that ZF–TET2 induced the highest level of DNA demethylation when compared to ZF–TET1 and ZF–TET3. Theirs was the only study to compare the TET enzymes directly; all other TET-associated demethylation tools have utilized TET1. More comprehensive assessment of the demethylation activities of different TET proteins may improve future design strategies.

**Fig. 3:**
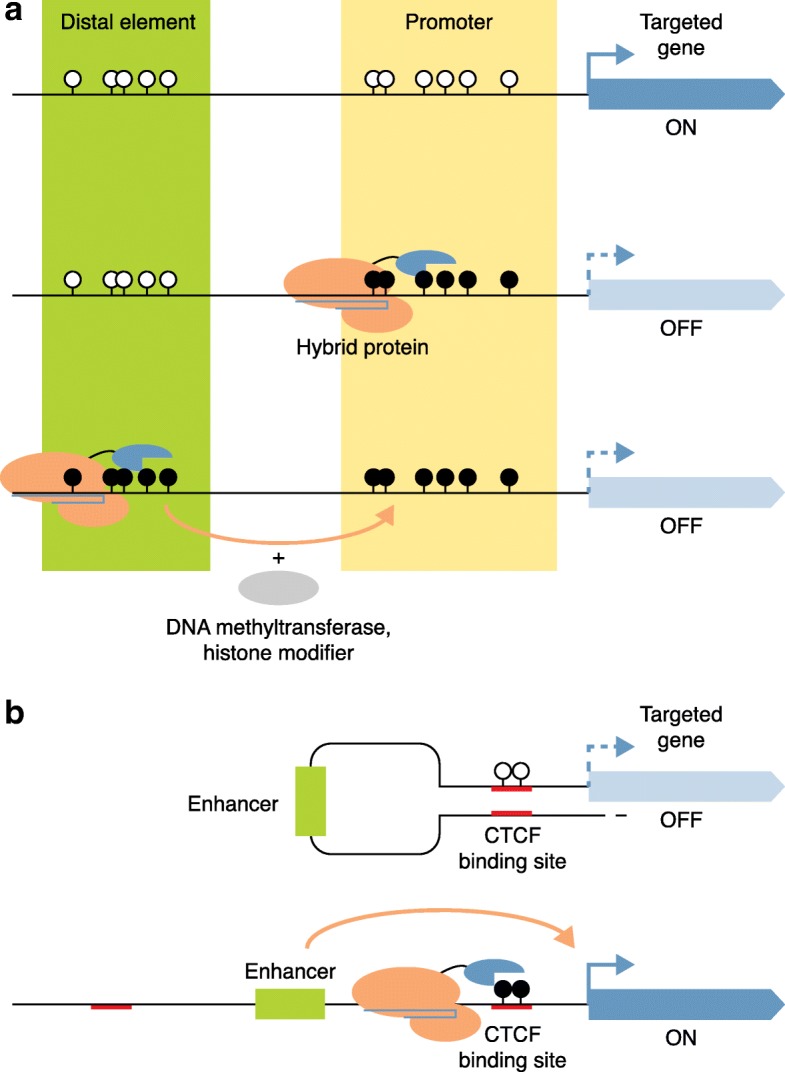
How targeted CpG methylation affects gene expression. **a** Direct methylation of a promoter to silence expression or to edit a distal element (such as an enhancer) in order to recruit endogenous DNA methyltransferase or histone modification to silence expression. **b** Targeted CpG methylation at CTCF-binding sites to open CTCF looping and thus activate gene expression. *Hollow* and *black-filled circles* indicate unmethylated and methylated CpG sites, respectively. *Red lines* indicate CTCF-binding sites

In 2016, several targeted CpG demethylation studies using CRISPR were published. Both transient and lentiviral-based stable methods of expressing the dCas9–TET1 fusion protein have been reported [40, 56]. Another study utilized a modified sgRNA by inserting two copies of bacteriophage MS2 RNA elements into the conventional sgRNA, facilitating the recognition and gathering of the catalytic domain of TET1 (TET1CD) [57]. To enhance TET1 recruitment and demethylation, Morita et al. [46] applied a dCas9–SunTag-based strategy by gathering scFv–TET1CD, achieving up to 90% demethylation in different cell types and in mouse fetuses. In addition to targeting promoters directly, this dCas9–TET1CD demethylation tool was also applied to demethylate the *MyoD* distal enhancer and thus to promote the myogenic reprogramming of fibroblasts [40] (Fig. 3a). Recently, the dCas9–TET1CD tool also was applied to demethylate CGG repeats in Fragile X syndrome-induced pluripotent stem (iPS) cells and reactivate the silenced *FMR1* by activating its promoter. Liu et al. [58] found that this induced reactivation was sustainable in a human–mouse chimeric model. Thus, this success indicates potential applications in examining the causality of disease-associated DNA methylation alterations and in the evaluation of future therapeutic consequences after targeted DNA demethylation.

Another novel study utilized the catalytic domain of *Arabidopsis* ROS1 5mC DNA glycosylase (ROS1CD), which directly excises 5mC and initiates its substitution for 5C, to rescue transcription of a methylation-silenced reporter gene. Parrilla-Doblas et al. [59] connected ROS1CD to the DNA-binding domain of yeast GAL4 and demonstrated a substantial decrease in the methylation level at targeted promoters, followed by increased transcription. Although current targeted demethylation tools have enabled the activation of silenced genes, additional studies and optimizations are still needed. These include more comprehensive off-target investigation and development of a better understanding of how the hybrid protein and DNA helix interact at the three-dimensional level.

## Assessing the role of methylation in transcription

In the past, DNA methylation was believed to regulate the transcriptome by repressing transcription [60]. Recent microarray and sequencing data suggest, however, that methylated DNA induces nucleosome assembly and reduces DNA accessibility, and that these processes play an essential role in silencing gene expression [61]. In order to test this concept, several targeted CpG methylation experiments were performed at promoters, where CpG sites usually have low levels of methylation. Both de novo methylation and gene repression were reported at desired loci [38–40]. Similarly, to increase our understanding of how demethylation affects gene expression, targeted demethylation was also applied at promoters or around transcription start sites (TSS). For example, a study using dCas9–TET1CD showed an increase in expression of the *BRCA1* gene of about two-fold in two human cell lines. This demethylation tool also induced the expression of green fluorescent protein (GFP), which was under the control of the fully methylated *Snrpn* promoter [56].

In addition to the targeting of local CpGs at promoters directly, other strategies have been applied to enhance methylation effects by recruiting exogenous DNMTs. One report showed that CRISPR-based DNMT3A–3 L enabled the recruitment of endogenous DNMT3A [44], resulting in extensively methylated regions. Another demonstrated that MS2-coated sgRNA gathers induced TET1CD and generates ~ 0.5 kb hypomethylation [57]. Further, the SunTag strategy has been reported to induce the assembly of scFv–DNMT3A [47] or scFV–TET1 [46], generating DNA hypermethylation over 1 kb or inducing up to 90% demethylation, respectively. These long-range strategies enable editing of the whole CGI or promoter region, and could potentially influence chromatin structure, including nucleosome placement and chromatin accessibility. Besides manipulating DNA methylation at promotors, targeted methylation of gene-body regions or other non-coding regions could be informative, but has not yet been explored. Recently, Su et al. [62] demonstrated that hypermethylation of gene-body regions in the homeobox genes is associated with higher levels of gene expression. Previous studies had shown that methylated CpGs at CTCF-binding sites (CBS) could alter chromatin looping and could thereby impact local gene expression. Liu et al. [40] and Lei et al. [48] used CTCF–ChIP to investigate CTCF anchoring after the induction of methylation at CBS. Both groups found that CTCF binding at the desired sites was significantly reduced, supporting the notion that DNA methylation blocks CTCF anchoring and thus alters looping formation. These observations are consistent with results from CBS deletion studies [63]. Thus, the induction of long-range hypermethylation or targeted hypermethylation at key elements that alter chromatin folding may be more effective than local CpG methylation in influencing transcription.

Despite these recent technological advances, the causal relationship between DNA methylation and transcription is complex and incompletely understood. Still, it is clear that the anchoring of a fusion protein to DNA can reduce gene transcription by blocking the initiation or elongation of RNA polymerase complexes [64]. Thus, some of the silencing observed in these studies may not necessarily result from DNA methylation and thus results should be interpreted cautiously. Further, another study suggested that histone methylation alone is insufficient to repress target genes stably. O’Geen et al. [65] showed that the deposition of repressive chromatin histone hallmarks H3K9me3 (H3K9 tri-methylation) and H3K27me3 did not necessarily correlate with decreased transcription, suggesting that both DNA methylation and histone marks contribute to governing transcription. These studies may explain why the downregulation of gene expression observed after targeted DNA methylation has to date been relatively modest [38–40].

Unlike DNA methylation, DNA demethylation is likely to be positively associated with transcription. We can therefore reasonably rule out the possibility that gene upregulation results from spatial occupancy, which usually negatively affects gene expression. Indeed, in the above experiments, the relevant control comprising a disabled catalytic domain failed to increase targeted gene expression. To examine possible long-range effects, Liu et al. [40] applied dCas–TET1CD to target *MyoD*, a distal enhancer located over 20 kb upstream of its TSS. Demethylation of the *MyoD* enhancer in mouse embryonic fibroblasts resulted in a moderate upregulation of expression and substantially facilitated myoblast conversion and myotube formation in the presence of 5-Aza. This study suggests that DNA methyltransferase inhibitors could be applied to enhance the effects of targeted demethylation editing. Further studies in induced pluripotent stem cells (iPSCs) from Fragile X syndrome (FXS) showed that targeted DNA demethylation recruited RNA polymerase II (Pol II) and generated histone H3K4me3 and H3K27Ac (H3K27 acetylation) modification at the FM R1 promoter. These modifications activated the expression of a previously silenced gene [58], supporting the concept that, at least in certain circumstances, DNA demethylation is sufficient to activate heterochromatic DNA and to rebuild an epigenetic landscape that allows active histone modifications.

## Profiling the distance of DNA methylation editing

CRISPR–Cas9 is known to provide very specific and precise cleavage of the DNA helix, generating a double-strand break between the third and fourth base pairs upstream of the PAM [66], but the optimal distance from the sgRNA anchoring site to the desired CpG targets for DNA methylation editing remains unknown. Because DNA methylation is performed by the enzyme fused to dCas9, the size of the fusion protein should influence the editing distance. Vojta et al. [38] fused DNMT3ACD (amino acids 602–912) to the C-terminus of dCas9 via a short nuclear localization signal (NLS)–Gly_4_Ser linker. After introducing the protein and guides into mammalian cells, pyrosequencing showed a 25–35 bp wide peak of methylation activity centered about 27 bp downstream of the PAM. Meanwhile, a minor methylation peak was observed about 25 bp upstream of the sgRNA binding sites. McDonald et al. [39] applied a similar tool, fusing DNMT3ACD (amino acids 598–912) to the C-terminal of dCas9 via a NLS–FLAG tag linker, and found that DNA methylation occurred within about 50 bp of the sgRNA-binding site. In order to increase the effect of editing methylation, Stepper et al. [44] fused an engineered DNMT3ACD–DNMT3L to the dCas9 via a 28-amino-acid linker (NLS included). The authors observed greater methylation activity at target sites when using this hybrid protein than when using the dCas9–DNMT3ACD tool, achieving a major methylation peak at about 25 bp downstream and a secondary peak at about 40 upstream of the PAM sites. Their summarized methylation profile presented a hypermethylation effect that occurred as far as ±400 bp away from sgRNA-binding sites. In addition, a dCas9–DNMT3A tool was also reported [40] to induce an average 25% increase in methylation within a 320-bp region of the *p16* promoter using a single sgRNA. Finally, Huang et al. [47] demonstrated ~ 4.5 kb of hypermethylated DNA at HOXA loci using dCas9–SunTag–DNMT3A, whereas in the same study, the intragenic *KLF4* loci showed CpG hypermethylation only within 300 bp of the sgRNA-binding sites (Table 1). These data indicate that factors in addition to physical constraints influence DNA methylation editing efficiency around specific targets. Further systematic studies are therefore necessary before we can change CpG methylation predictably at desired sites.

**Table 1:** Summary table of TALE and CRISPR based targeted methylation tools

Strategy	Delivery	Efficacy	Duration	Locus	Peak site	Editing range	Target	Off-targets	Features	Refs
TALE-DNMT3ACD-3 L	Transfection Transduction	12–15% 17%	2 days 4 days	CDKN2A	n.a.	~ 700 bp	CpG	At nearby regions (Targeted mePCR)	First TALE-DNMT3 A study (2015)	[33]
TALE-CIB1; DNMT3A/TET1 CD-C R Y2	Transfection	~ 10%	~ 5 days	Ascl 1	n.a.	n.a.	CpG	n.a.	Epigenome editing by optogenetics (2017)	[34]
dCas9-DNMT3ACD	Transfection	~ 35% ~ 43%	8 days 3 days	BACH2 CDKN2A	−27 bp, + 25 bp	< 100 bp	CpG	Off-targets observed using unspeci_c sgRNAs	First dCas9-DNMT3 A studies (2016)	[38, 39]
dCas9-DNMT3A	Transduction	~ 12% ~ 25%	3 days 3 days	*Gapdhsnrpn* *CDKN2A*	n.a.	n.a.	CpG	Not detectable (dCas9 ChIP-seq)	Targeted methylation alters CTCF looping and local genes expression (2016)	[40]
TALE/dCas9-KRAB, DNMT3ACD, DNMT3L	Transduction	~ 80%	30 days	*B2M*	n.a.	~ 3 kb	CpG	Not detectable (MeDIP-seq)	Inheritable silencing of endogenous genes (2016)	[43]
dCas9-DNMT3ACD-3 L	Transfection	~ 30%	5 days	*TFRC* *CXCR4*	−40 bp, + 25 bp	~ 800 bp	CpG	Mild off-target observed	More e_cient than dCas9- DNMT3ACD (2016)	[44]
dCas9-Sun Tag-DNMT3A	Transduction	~ 70% ~ 45%	30 days	*HOXA5* *DLX1*	n.a.	~ 4.5 kb ~ 500 bp	CpG (CpH)	Not detectable (RRBS; WGBS)	Long range methylation Non-CpG methylation (2017)	[47]
dCas9-Sun Tag-DNMT3ACD	Transfection	~ 15%	4 days	*UNC5C*	n.a.	n.a.	CpG	Not detectable (TSC-bs-seq)	Modular Sun Tag- DNMT3ACD reduces off-target events (2018)	[62]
dCas9-MQ1 ^147^	Transfection	*60%	24 h	*HOXA5* *HOXA4* *RUNX1*	−24-26 bp	n.a.	CpG	Not detectable (RRBS)	In vivo application using zygote injection (2017)	[54]
dCas9-split M.SssI	Transfection	*50%	2 days	*SALL2*	−22-23 bp	8–25 bp	CpG	Not detectable (Targeted mePCR)	Split catalytic domain for higher speci_cty (2017)	[48]

*Maximum DN A methylation at a CpG

Employing prokaryotic DNA methyltransferase as an effector domain excludes the probability of recruiting endogenous DNMTs and forming a DNMT3A–DNMT3L complex at desired sites. In theory, this design could exhibit a relatively clear correlation between sgRNA-binding sites and modified CpGs. Lei et al. [48] used dCas9–MQ1^Q147L^ to obtain a specific methylation peak at about 20–30 bp downstream of the sgRNA-binding site, although a secondary peak upstream was also observed. Another prokaryotic tool split M.SssI into two parts, MN and MC [49], then fused MC to dCas9 via a 15-amino-acid flexible linker (GGGGS)3. This tool yielded methylation only in a region located about 8–25 bp downstream of the sgRNA-binding site, with the methylation peak occurring 12 bp and 22–23 bp away from the PAM. In addition to the editing periodicity mentioned above, this tool exhibited methylation strand-specific differences by editing the strand that was in *trans* better than the one in *cis* [49]. To date, reports of targeted demethylation have omitted similar profiling of editing distance, but these questions should be addressed in order to ensure the appropriate application of these tools (Table 2).

**Table 2:** Summary table of TALE and CRISPR based targeted demethylation tools

Strategy	Delivery	Efficacy	Duration	Locus	Peak site	Editing range	Target	Off-targets	Highlights	Refs
TALE-TET1CD, TALE-TET1	Transfection	~ 15%	4 days	*KLF4* *HBB*	n.a.	n.a.	CpG	Off-targets from unspeci_c binding	First TALE-TE T study (2013)	[53]
dCas9-TET1CD	Transfection	~ 15%	24 h	*BRCA1*	n.a.	n.a.	CpG	Not detectable in LINE1 elements	First dCas9-TE T study (2016)	[56]
dCas9-TET1CD, MS2-TET1CD	Transfection	~ 15%	4 days	*RANKL* *MMP2* *MAGEB2*	n.a.	~ 500 bp by multi-sgRNAs	CpG	Not detectable at sgRN A similar sites	MS2 coated sgRNA (2016)	[57]
dCas-TET1CD	Transduction	~ 26%	3 days	*Dazl-Snrpn*	n.a.	n.a.	CpG	Minimal o ff-target on methylation and expression using dCas9-ChIP-BS-seq and RNA-seq	Induce an active chromatin status for promote r. Sustainable in a human/mouse chimeric model (2016)	[40, 58]
dCas9-Sun Tag-TET1CD	Transfection	~ 80%	2 days	*Gfap* *H19*	n.a.	200–1000 bp	CpG	Not detectable (WGBS; RNA-seq)	Long range demethylation. In vivo delivery by in utero electroporation (2016)	[46]
Gal4-ROS1 CD	Transfection	n.a.	2 days	Reporter plasmid	n.a.	n.a.	CpG	n.a.	Direct removal of 5mC without hydroxymethylation (2017)	[59]

The studies discussed above suggest that dCas9 with a single methyltransferase fusion protein acts in a relatively local manner, although secondary regions of methylation induction were frequently reported elsewhere. Whether these regions of methylation induction are caused by off-target effects remains unclear. Of note, very recent studies using dCas9–DNMT3A tools [54, 55, 58] revealed widespread off-target activity when analyzing the genome comprehensively, raising concerns about the interpretation of limited off-target effects in earlier studies in which the methylation analysis was more focused. On the other hand, results obtained using tools that utilize multimerization, such as SunTag, show long-range methylation activity. Yet, there is little direct experimental evidence to show whether such reported long-range methylation results from multimerization features. The contribution to these results from other factors, including the time of induction (which varies from 24 h to 40 days), delivery strategies (transient expression or lentiviral transduction), expression strength (inducible or continuous), and global binding specificity, cannot be ruled out. In addition, DNA structure, histone modification, and DNA topology all play important roles in shaping the methylome. Therefore, it is currently challenging to predict the pattern of de novo methylation induced by a particular tool at a particular site. More detailed structural insight into these tools and how they interact with the DNA helix is necessary to address these remaining questions.

## Targeted methylation/demethylation tools induce off-target effects

Off-target effects are always of paramount concern when manipulating the genome with any exogenously introduced tool. Whether or not there are clear and immediate biological consequences, the off-target profiles of these hybrid proteins must be understood before these tools are applied widely for research or therapeutic purposes. There are two types of dCas9–MTase-induced off-target effects: 1) misrecognition of the dCas9–sgRNA complex, and 2) unintended methylation by the DNA methyltransferase. To examine the first, a genome-wide dCas9 chromatin immunoprecipitation and high-throughput sequencing (ChIP-seq) experiment illustrated that the number of off-target sites varied from ~ 10 to more than 1000 depending on the sgRNAs used [67]. However, in a combined dCas9 ChIP-seq and bisulfite sequencing experiment, Liu et al. [40] showed that even at those sites with the highest predicted likelihood of off-target effects, dCas9–DNMT3A only induced marginal methylation relative to the higher DNA methylation at the designed loci, suggesting that any non-specific binding may cause minimal off-target effects. Other studies applied genome-wide sequencing technologies, including reduced representation bisulfite sequencing (RRBS) and whole genome bisulfite sequencing (WGBS), to assess potential side effects of various methylation tools and reported no detectable off-target hypermethylation (dCas9–MQ1^Q147L^ and dCas9–SunTag–DNMT3A/TET1) [47, 48]. Similarly, few off-target effects have been reported with demethylation tools. For example, no obvious off-target effects were observed in a dCas9–TET1CD study using pyrosequencing of LINE1 elements [56], and no off-target methylation was detected in the SunTag study using both WGBS and RNA-seq [46]. Another study from Liu et al. [58] showed minimal off-target methylation and expression using anti-dCas9 ChIP–BS-seq and RNA-seq (Table 2).

Nevertheless, studies using the dCas9–DNMT3ACD tool without sgRNA or with non-specific sgRNA did report obvious off-target methylation [39, 47, 48]. A mild methylation increase was also reported at some of the top-predicted off-target sites that were identified on the basis of similarity to the sgRNA [44]. A recent genome-wide study tracking dCas9–DNMT3ACD footprints uncovered the presence of pervasive global off-target methylation in mouse embryonic stem cells (mESC) with initial low-level methylation, as well as in somatic cells, regardless of whether or not sgRNA was present [55]. This comprehensive study argued that the ubiquitous non-specific activities of dCas9 might have negative implications for dCas9-fused epigenetic editing tools. Another study demonstrated that modular dCas9–SunTag–DNMT3ACD could overcome the ubiquitous off-target activity associated with DNMT3ACD [54]. This study echoed the results of Huang et al. [47], who used WGBS to demonstrate that the dCas9–SunTag–DNMT3A tool had a minimal effect on the global DNA methylome. Nevertheless, off-target analysis has been insufficient in many studies to date. In vertebrates, around 60–80% of CpG exist in a highly methylated status, and only a relatively small fraction remain in an unmethylated or partially methylated state [68]. Therefore, the global effects of methylation may not be discerned easily. The dynamic state of the methylome poses an even greater challenge. Unlike the sequence of DNA, DNA methylation is changeable and can be altered during cellular proliferation and differentiation. Thus, systematic comparisons of the potential off-target effects of these tools are not yet well-established. Both local and global off-target assessments should be included in future studies.

## Tools to induce non-CpG DNA methylation

Non-CpG methylation (CpA, CpT, and CpC) is highly enriched in embryonic stem cells [2, 69, 70], iPSCs [71], and adult brain tissue [1] but is scarce in most other differentiated cell types [3]. In neuronal tissue, DNMT3A has been shown to establish non-CpG methylation [70, 72, 73]. As DNMT3A is read by MECP2 [74, 75], the accumulation of non-CpG methylation is correlated with DNMT3A expression in the brain [1]. Although the role of non-CpG methylation in gene expression has been studied for a decade [70], and non-CpG methylation has been shown to accumulate with synaptic development and synaptic density [1], the relationship of non-CpG methylation with neuronal development remains largely unknown.

Huang et al. [47] utilized the dCas9–SunTag–DNMT3A system to methylate the HOXA5 locus and found that not only CpGs but also many non-CpGs across this region were methylated, reinforcing the finding that DNMT3A is responsible for non-CpG methylation. Furthermore, in a genome-wide characterization of dCas9-methyltransferase footprints, Galonska et al. [55] showed that the expression of full-length DNMT3A led to both CpG and non-CpG methylation, but that constructs that contained only the catalytic domain of DNMT3A resulted in methylation of CpGs only [55]. These results suggest that the regulatory domains of DNMT3A are essential for establishing non-CpG methylation and potentially explain why previous dCas9–DNMT3ACD tools did not induce non-CpG methylation. Recent reports offered structural and mechanistic insights into how DNMT3A recognizes its substrates and conducts its enzymatic activity [76]. For example, Zhang et al. [76] showed that the Arg836 residue of DNMT3A is critical for determining the preference of CpGs over non-CpGs. Using in vitro biochemical and cell-based assays, these authors found that the Arg836Ala mutant (DNMT3A^R836A^) had higher non-CpG methylation activity whereas CpG methylation activity remained unchanged. To date, no targeted non-CpG demethylation study has been reported.

In summary, findings have shown that dCas9–SunTag–DNMT3A can induce targeted non-CpG methylation and that specific mutations in DNMT3A can lead to higher non-CpG methylation. In order to better understand the roles of non-CpG methylation in gene expression and neuronal development, further studies are necessary to test whether non-CpG methylation could be induced without changing CpG methylation.

## Current challenges for manipulating DNA methylation to regulate transcription

To date, transiently or stably altering gene expression has been necessary in order to resolve biological questions. Transient regulation methods such as short hairpin RNA (shRNA) or small interfering RNA (siRNA) cannot induce persistent effects, whereas genome-editing approaches such as CRISPR or stable-expression approaches, including lentiviral or retroviral, can cause permanent alterations in genetic sequence. Epigenetic editing tools, on the other hand, have the potential to regulate transcription consistently during proliferation without introducing a genetic sequence change. However, current targeted methylation tools remain in preliminary stages of development and require further improvement before they can uncover the causal relationship between epigenetic marks and the regulation of transcription.

The fact that CpG methylation and transcriptional repression are imperfectly correlated is a significant obstacle for current methylation editing tools. In addition, there can be hundreds of CpGs in one single promoter and whether these CpGs contribute equally to repression or whether some have more importance in controlling gene expression remains unclear. If ‘key CpGs’ exist, how and where to identify them remains an open question. Thus, in order to alter gene expression, most sgRNAs that have been used to induce targeted methylation or demethylation were designed to be located near the TSSs of the genes whose expression is to be changed. Furthermore, numerous studies have shown that some CpGs are more efficiently hypermethylated or demethylated than others, suggesting that the epigenetic status of a particular CpG may be predetermined and maintained by histone modification, chromosome accessibility, or maybe the DNA sequence itself. Systematic studies of the impact of methylation on specific CpGs could reveal fundamental regulatory principles and will facilitate the future design of tools for effective silencing.

Several studies have applied tools to target smaller elements, including CTCF-binding sites and transcription factor binding sites. These precise editing tools may be able to indicate a relatively clear correlation between induced epigenetic modification and expression changes (Fig. 3b). Although one previous study illustrated that the methylation of a single CpG in the *IL6* promoter affected *IL6* gene regulation [77], a successful strategy to identify the most relevant CpG sites in other loci accurately requires further study. To date, most CRISPR–dCas9 chimeric proteins employ natural *Streptococcus pyogenes* Cas9 (SpCas9) [78], the binding domain of which recognizes an NGG PAM sequence. While broadly deployed, this PAM requirement constrains the guide design within a given region, limiting some strategies involving targeted methylation and demethylation tools. Other Cas9 proteins, including engineered SpCas9 [79], *Staphylococcus aureus* Cas9 (SaCas9) [80], and Cpf1 [81] could be employed in the future to offer broader sgRNA-binding options.

Another challenge for targeted DNA methylation editing is estimating the level of CpG methylation required to repress gene expression. Large-scale deep sequencing from patient and control samples has shown that hypermethylation occurs at silenced loci, suggesting that complete methylation or demethylation is needed to modulate gene expression fully. Unfortunately, most of the DNA methylation editing tools described to date are unable to efficiently methylate or demethylate whole CGI, probably explaining, at least in part, their moderate effects on gene expression. For example, Lei et al. [48] were able to partially hypermethylate the *Igf2/H19* imprinting loci in mouse embryos, but this change had no apparent impact on mouse body weight. Similarly, Liu et al. [40] found that partial demethylation of the *MyoD* enhancer was insufficient to induce myotube formation without 5-Aza treatment. At a different locus, however, the same demethylation tool was able to demethylate the targeted CGG repeat efficiently, resulting in demethylation of the *FMR1* promoter CGI, stable activation of *FMR1* transcription, and re-establishment of active histone modifications [58]. It is likely that the repeat sequence at the desired site enriched the fusion protein and enhanced the demethylation effect. Thus, strategies to increase the level of methylation/demethylation in order to induce more obvious transcriptional changes should be the focus of future studies.

How the forcible hypermethylation or demethylation status is maintained during cell proliferation and differentiation remains largely unknown. Vojta et al. [38] found that the greatest methylation effect occurred about 7 days after transfection in 6-week time-dependent experiments. Similar patterns have been reported in other methylation and demethylation studies [48, 53], indicating that transient epigenetic editing approaches for CpGs may not deliver durable effects. A recent dCas9–DNMT3ACD study that induced methylation in an edited mESC showed that methylation levels decreased within 7 days post-transfection not only at targeted but also at most off-target sites [55]. Although it is clear that demethylation occurs over time, further investigation is required to understand whether it occurs through an active or a passive mechanism. Another study measured how induced DNA methylation changes after cell-cycle arrest [82] and found that DNA replication was not required for loss of methylation, which strongly implies active removal mediated by TET enzymes and accessory factors. The identity of the signal or signals that recruit TET enzymes to the induced methylation site remains unknown.

Aside from current applied prokaryotic and mammalian DNA methyltransferases, other hypermethylation approaches should also be explored and assessed. For example, the KRAB domain has been widely applied for transient transcriptional repression. KRAB recruits repressive histone modifiers, including KRAB-associated protein 1 (KAP1), histone methyltransferases SETDB1, the nucleosome remodeling complex (NuRD), and heterochromatin protein 1 (HP1). Previous ZF–KRAB studies in mouse early embryogenesis demonstrated KRAB-mediated repression results in irreversible gene silencing through promoter hypermethylation if it acts before implantation (around day E3.5), which is the key time point when mouse zygotes start to rebuild genome-wide methylation [83, 84]. Despite the fact that the molecular mechanism underlying this observation remains unknown, these results indicate that KRAB-induced DNA methylation could escape from zygote-wide demethylation after fertilization and thus could be maintained following embryogenesis. Thus, the KRAB domain has promising utility for inducing targeted methylation in very early embryos. Another intriguing study showed that the insertion of a CpG-free DNA fragment induced de novo methylation of the entire targeted CGI in human pluripotent stem cells (hPSCs) [85]. In this study, the methylation level appeared to be maintained following the removal of the CpG-free fragment, extensive passaging, and differentiation. This stable methylation led to the correction of irregular imprinting in Angelman syndrome-derived human iPSCs. Although the underlying molecular mechanisms remain elusive, the above studies suggest additional strategies to deploy stable DNA methylation.

Finally, successful epigenetic regulation of an intended gene depends on the precise addition or removal of epigenetic marks. Many current targeted methylation and demethylation expression cassettes are under the control of a strong expression promoter. The side effects of the long-term and consistent expression of methylation or demethylation proteins have not yet been examined. Thus, the delivery of targeted methylation or demethylation proteins without vectors may decrease potential off-target risks.

## Conclusions

Current tools to induce targeted methylation and demethylation may be able to improve our understanding of the role that DNA methylation plays in regulating gene expression. Studies to profile the effects of these tools systematically will shed light on how methylation is altered during biological processes. In the future, using these tools to establish direct links between transcriptional regulation and DNA methylation status will enable us to decipher the precise role of epigenetic modification in health and disease and will increase our overall understanding of the human genome.

## Abbreviations

CBS: CTCF-binding site
CGI: CpG island
ChIP-seq: Chromatin immunoprecipitation and high-throughput sequencing
dCas9: Catalytic dysfunction Cas9
DNMT: DNA methyltransferase
iPSC: Induced pluripotent stem cell
KRAB: Krupple-associated box
mESC: Mouse embryonic stem cell
NLS: Nuclear localization signal
PAM: Protospacer adjacent motif
RHD: Rel-homology domain
RRBS: Reduced representation bisulfite sequencing
sgRNA: Single guide RNA
shRNA: Short hairpin RNA
siRNA: Small interfering RNA
TALE: Transcription activator-like effector
TDG: Thymine DNA glycosylase
TET: Ten-eleven translocation
TSS: Transcription start site
WGBS: Whole genome bisulfite sequencing
ZF: Zinc-finger
